# Radon exposure is rising steadily within the modern North American residential environment, and is increasingly uniform across seasons

**DOI:** 10.1038/s41598-019-54891-8

**Published:** 2019-12-03

**Authors:** Fintan K. T. Stanley, Jesse L. Irvine, Weston R. Jacques, Shilpa R. Salgia, Daniel G. Innes, Brandy D. Winquist, David Torr, Darren R. Brenner, Aaron A. Goodarzi

**Affiliations:** 1 Robson DNA Science Centre, Charbonneau Cancer Institute, Departments of Biochemistry & Molecular Biology and Oncology, Cumming School of Medicine, University of Calgary, Calgary, Alberta Canada; 2 Radon Environmental Management Corporation, Vancouver, British Columbia Canada; 3 College of Medicine, Community Health and Epidemiology, University of Saskatchewan, Swift Current, Saskatchewan Canada; 4 Public Health Physicians of Canada and Saskatchewan Health Authority, Saskatchewan, Canada; 5 Robson DNA Science Centre, Charbonneau Cancer Institute, Departments of Cancer Epidemiology & Prevention Research and Community Health Sciences Cumming School of Medicine, University of Calgary, Calgary, Alberta Canada

**Keywords:** Cancer prevention, Environmental impact, Lung cancer, Risk factors

## Abstract

Human-made buildings can artificially concentrate radioactive radon gas of geologic origin, exposing occupants to harmful alpha particle radiation emissions that damage DNA and increase lung cancer risk. We examined how North American residential radon exposure varies by modern environmental design, occupant behaviour and season. 11,727 residential buildings were radon-tested using multiple approaches coupled to geologic, geographic, architectural, seasonal and behavioural data with quality controls. Regional residences contained 108 Bq/m^3^ geometric mean radon (min < 15 Bq/m^3^; max 7,199 Bq/m^3^), with 17.8% ≥ 200 Bq/m^3^. Pairwise analysis reveals that short term radon tests, despite wide usage, display limited value for establishing dosimetry, with precision being strongly influenced by time of year. Regression analyses indicates that the modern North American Prairie residential environment displays exceptionally high and worsening radon exposure, with more recent construction year, greater square footage, fewer storeys, greater ceiling height, and reduced window opening behaviour all associated with increased radon. Remarkably, multiple test approaches reveal minimal winter-to-summer radon variation in almost half of properties, with the remainder having either higher winter or higher summer radon. This challenges the utility of seasonal correction values for establishing dosimetry in risk estimations, and suggests that radon-attributable cancers are being underestimated.

## Introduction

Lung cancer is the 6^th^ leading overall cause of death and the foremost cause of cancer death in the world. It is understood to be predominantly triggered by chronic inhalation of tobacco smoke and/or radioactive radon (^222^Rn) gas, often coupled with underlying genetic predispositions^1–6^. Radon is a primary cause of lung cancer in never smokers and the second leading cause in smokers, encompassing an estimated 3–20% of lung cancer deaths worldwide^7,8^. Gaseous radon isotopes arise from decaying uranium, thorium and radium-containing minerals in bedrock, surficial materials and groundwater that are prevalent globally^1,9^. First order estimations of radon potential have been classified previously based on radiometric data derived from uranium and thorium radionuclide content of bedrock lithology, surficial materials, groundwater, structures and anthropogenic activity^10,11^. There are positive correlations in uranium (ppm), estimated from airborne radiometric and direct indoor measurements, that additionally account for permeability factors in the assessment of radon mobility in surficial materials (i.e. groundwater history)^12^. Although arising naturally, radon and radon-derived ‘daughters’ (including ^214^Po, ^218^Po) can concentrate within the built environment to levels typically not observed in nature. Thus, hazardous radon exposure is largely an anthropogenic environmental health issue.

Radon synergizes with lung carcinogens such as tobacco smoke to multiply lung cancer risk^13,14^. However, unlike tobacco use, radon inhalation is not addictive and effective testing and mitigation techniques exist^15^. Thus, radon exposure represents a readily preventable cause of the most lethal and common cancer type, and is a priority area of public health intervention and cancer prevention. Decaying ^222^Rn emits alpha particle ionizing radiation, severely damaging DNA in such a way that is almost impossible for our cells to repair without introducing genetic errors^16^. Such errors trigger ‘genomic instability’, a self-propagating cycle of DNA alteration that drives cancer formation^2^. Further to this, approximately 1 in 30 adult humans display radiation sensitivity, meaning that (compared to the average) they over-respond to ionizing radiation exposure leading to moderate to severe health effects including morbidities, mortality and/or increased risk of cancer^17–20^. The *International Agency for Research on Cancer* lists radon as a category 1 carcinogen, meaning it is unequivocally known to cause human and animal cancers^1^. Ionizing radiation such as alpha particle radiation is measured in Becquerels (Bq) that represents one radioactive decay event per second. A 16% increase in relative lifetime risk of lung cancer is measurable per ≥100 Becquerel/m^3^ (Bq/m^3^) chronic radon inhalation^1,21,22^.

Historically, radon exposure is thought to be increased in cold climate regions where populations predominantly occupy closed indoor air environments long periods of the year to avoid adverse meteorological conditions. However, climate change and growing adoption of air conditioning across all regions may alter this 20^th^ century norm. It is estimated that the average North American spends 86.9% of their lives indoors^23^, meaning that analyzing the modern built environment is crucial for understanding exposure to many carcinogens. There are many regions of high radon potential on Earth, although this does not mean that all buildings in those areas contain unsafe radon levels^8,15^. Indeed, there are three factors needed to incur hazardous radon exposure: (i) a rich geologic source and pathway (upwards) for radon, (ii) environmental design metrics that actively draw up and concentrate radon and (iii) essential or elective human behaviour that prolongs exposure or increases radon concentrations. These latter two variables are potentially modifiable and are of interest in terms of exposure reduction.

Establishing historic and ongoing radon exposure represents significant ‘exposome’ information, similar to documenting smoking history^24^. Such information is important for early cancer detection programs, harm reduction^25^ and is also of interest to define best practice within scenarios such as business licensing, rental leasing, real estate transactions or home inspections. Thus, establishing the contextual (geographic, seasonal and environmental) effectiveness of distinct radon testing method(s) for decision-making is also important. Motivated by this, we measured household radon across a large North American area of high radon potential encompassing ~5.45 million humans spread across 1,313,748 km^2^. Radon dosimetry data was coupled to geospatial analysis, an interrogation of how built environment metrics and associated behaviours correlate with radon levels and, within a subset of regional buildings, an evaluation of multiple modalities of radon testing.

## Results

### Radon potential and domestic exposure in North America

**Figure 1:**
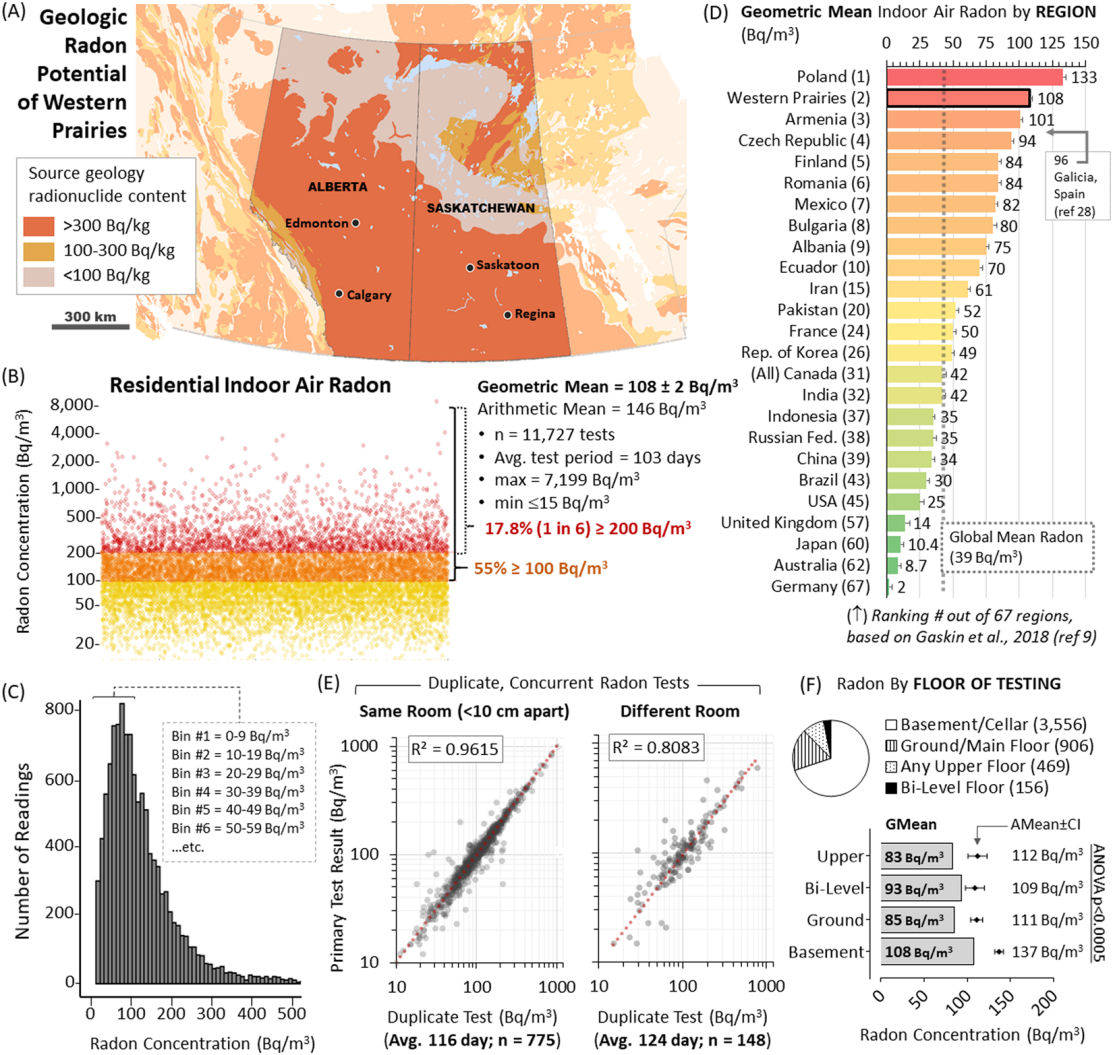
Radon Potential and Domestic Exposure in North American Prairies. Panel A: Geological radon potential map of the North American prairies highlighting Alberta and Saskatchewan. Orange regions contain >300 Bq/kg radon-generating geologic material; yellow contains 100–300 Bq/kg and pale grey-yellow contains <100 Bq/kg. Panel B: Domestic indoor air radon concentrations from all buildings tested within the area highlighted in (A). Yellow dots = 0–99 Bq/m^3^; Orange dots = 100–199 Bq/m^3^; Red dots ≥ 200 Bq/m^3^. All dots are 50% transparent to indicate data densities. Panel C: Histogram of data distribution binned into increments as indicated. Panel D: Geometric mean radon of the Western Prairies from this study, relative to levels documented by previous national studies and summarized in^9^. Panel E: Concurrent duplicate 90 + day radon tests plotted against each other (50% transparent black dots) with linear regression (red dotted line). Left graph shows duplicates placed <10 cm apart, right graph shows duplicates in a different room of the same building. Panel F: Pie chart shows distribution of reporting. Graph shows geometric mean radon (grey bars) and arithmetic mean radon with 95% confidence intervals (black diamonds with bars) by floor of test placement. ANOVA analysis outcomes indicate significance.

Geochemical composition of glacial tills (including outwash deposits, lacustrine clays, conglomerates, etc.) and derived soils can closely compare with local bedrock units and, as such, allows radon potential assessment^26^. Using this, we analyzed the radon potential of the Western North American Prairie Region using the US Geological Survey Data Series 424 as a base^27^. This method indicated the majority of the survey area contained geologies with greater than 300 Bq/kg of radon generating radionuclides (Fig. 1A). Thus, based on population density, survey region residents predominantly occupy areas of uniformly high geologic radon potential. The total radon dosimetry dataset encompasses 11,727 residential long term alpha track radon tests conducted between 2010–2018 in Alberta (AB) and Saskatchewan (SK), of which 55% (n = 6,257) were ≥100 Bq/m^3^ and 17.8% (n = 2,086) were ≥200 Bq/m^3^, the maximum tolerated exposure limit for Canada (Fig. 1B,C). The geometric mean for all tests was 108 Bq/m^3^ (arithmetic mean 146 Bq/m^3^), equivalent to 2.92 pCi/L (a non-SI unit commonly used in the USA) or, based on ICRP calculations, an annual adult lung equivalent radiation dose of 5.07 mSv/year. The median test duration was 103 days and 91% were deployed from October-April. Comparison to global radon levels recently compiled by Gaskin *et al*.^9^ (and accounting for other studies^28^) indicates the 1,313,748 km^2^ survey region encompasses one of the most radon-exposed large populations mapped to date (Fig. 1D). Concurrent duplicates confirmed test precision, with r^2^ = 0.962 for duplicates placed <10 cm apart and r^2^ = 0.808 for duplicates located in a different room but within the same building (Fig. 1E). Tests exposed to known quantities of radon demonstrate accuracy, with r^2^ = 0.996 (Supplementary Fig. 1A). There were no significant differences in radon levels reported by different room types (F (3, 5046 = 1.67, p < 0.17)) (Supplementary Fig. 1B). There were no significant differences in mean radon by the study year of testing (Supplementary Fig. 1C). Radon levels were statistically higher when the test device was placed on the basement/cellar level (F (4, 5063 = 8.20, p < 0.0001)), as compared to the main/ground and/or upper floors, encompassing a ~13% reduction in mean radon when comparing basement level to any upper floor (Fig. 1F). These data permitted the calculation of a test floor correction value of 1.2 between basement/cellar and any upper level. Applying this to normalize all readings to the lowest floor of testing, the overall level for the region was a geometric mean of 111 Bq/m^3^ (arithmetic mean = 150 Bq/m^3^).

### North American short versus long term radon test precision across seasons

**Figure 2:**
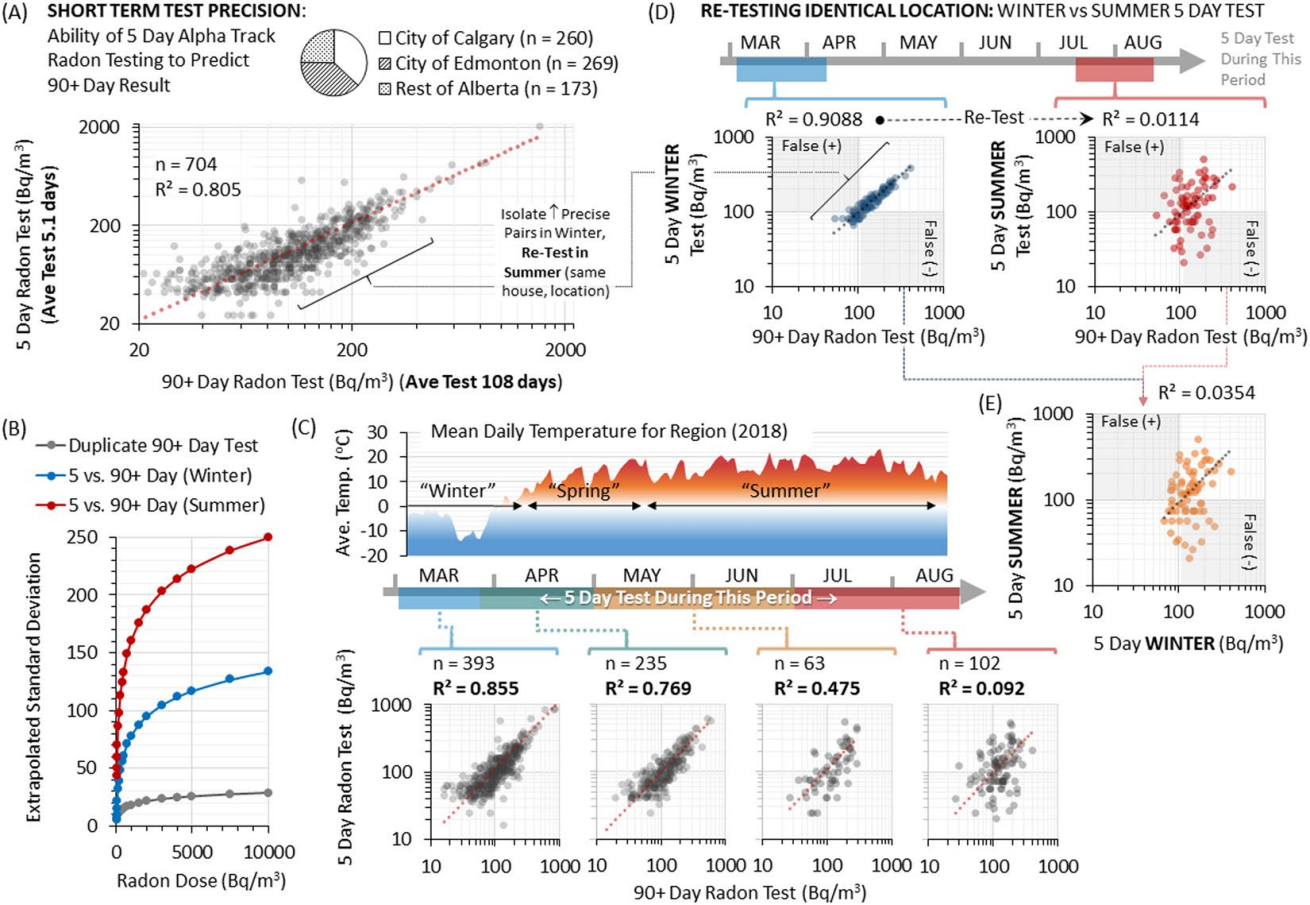
Radon test precision by duration and season of data capture. Panel A: Pie chart shows distribution of data reporting by city / region. 704 short term (5 day) alpha track radon tests were deployed <10 cm apart from (and in the latter 5 days of) a 90 + day winter alpha track test. Data points were plotted against each other (50% transparent black dots to show data density) with linear regression (red dotted line). Panel B: Standard deviations (SD) were calculated for concurrent 5 and/or 90 + day winter alpha track radon tests, using the data in Figs. 1E and 2A and as outlined in Supplementary Fig. 1. SD were extrapolated for radon doses up to 10,000 Bq/m^3^ for duplicate winter 90 + day tests, 5 versus 90 + day winter tests or 5 day summer tests versus 90 + day winter tests. Panel C: Upper graph indicates mean daily temperatures from March to August of 2018 for the survey region (Alberta), demarcating seasons. Lower graphs show the dataset from (**A**) subdivided by the specific date of the 5 day alpha track radon testing window, as indicated. Data points were plotted against each other (50% transparent black dots to show data density) with linear regression (red dotted line). Panel D: 100 × 5 day winter alpha track tests from (**A**) showing strong agreement with 90 + day winter tests were selected, and a second 5 day alpha track was deployed in the identical location in the same building during summer months, as indicated. Data points from 5 day winter or summer tests were plotted against the 90 + day winter test result (50% transparent blue (winter) or red (summer) dots to show data density) with linear regression (dotted lines). Panel E: The 5 day winter and summer radon data from (**E**) were plotted against one another as in (**D**).

To examine the influence of test duration on outcome, we deployed 704 short term alpha track radon test devices for the latter 5 days of the standard 90 + (winter) day test period used in this study; long and short term tests were placed < 10 cm apart (i.e. same room, same building, same time). Tests were restricted to AB, and split three-ways between two major population centres (Calgary and Edmonton) and the more rural remainder of the region (Fig. 2A). The 5 day tests predicted 90 + day counterparts 80% of the time (r^2^ = 0.805), noticeably less than r^2^ = 0.962 for 90 + day test duplicates (Fig. 1E). Per radon dose, standard deviation (SD) was greater between short and long term readings versus long term test duplicates (Fig. 2B, Supplementary Fig. 1D–G). Short term test precision displayed progressively decreasing r^2^ and increasing SD the further into warmer temperature months that short term tests took place (Fig. 2C). To interrogate this further, we isolated 100 pairs of 90 + and 5 day tests showing strong agreement (r^2^ = 0.909) in winter (March) and performed another 5 day test in the identical location in summer (July-August), finding r^2^ = 0.011 for a 5 day summer test versus a winter 90 + day test, or r^2^ = 0.035 when compared to a 5 day winter test (Fig. 2D,E). This indicates that short term radon tests display a 96–99% imprecision (i.e. failure) in predicting radon levels between seasonal extremes. These trends were consistent across the region and by building year of construction (Supplementary Fig. 1H).

### Geospatial analysis of the radon exposure by region

**Figure 3:**
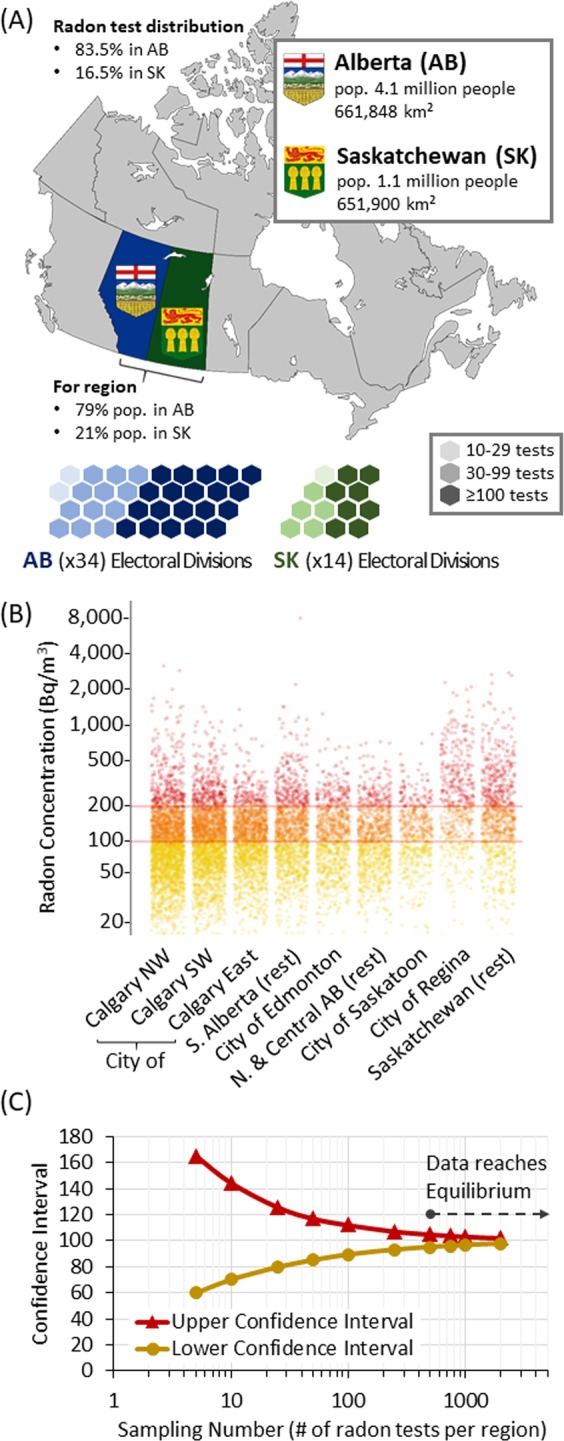
Geospatial Analysis of the Radon Exposure by Region. Panel A: Administrative map of Canada highlighting survey region provinces, relative population densities relative to study cohort distribution. Cartogram representing the federal electoral divisions in Alberta (blue) and Saskatchewan (green), color-coded by number of radon tests per division. Panel B: Domestic indoor air radon concentrations for buildings split by cities and region. Yellow dots = 0–99 Bq/m^3^; Orange dots = 100–199 Bq/m^3^; Red dots ≥ 200 Bq/m^3^. All dots are 50% transparent to indicate data densities. Panel C: Upper and lower 95% confidence intervals from bootstrapped mean estimates of radon concentration plotted against the resampling size.

**Figure 4:**
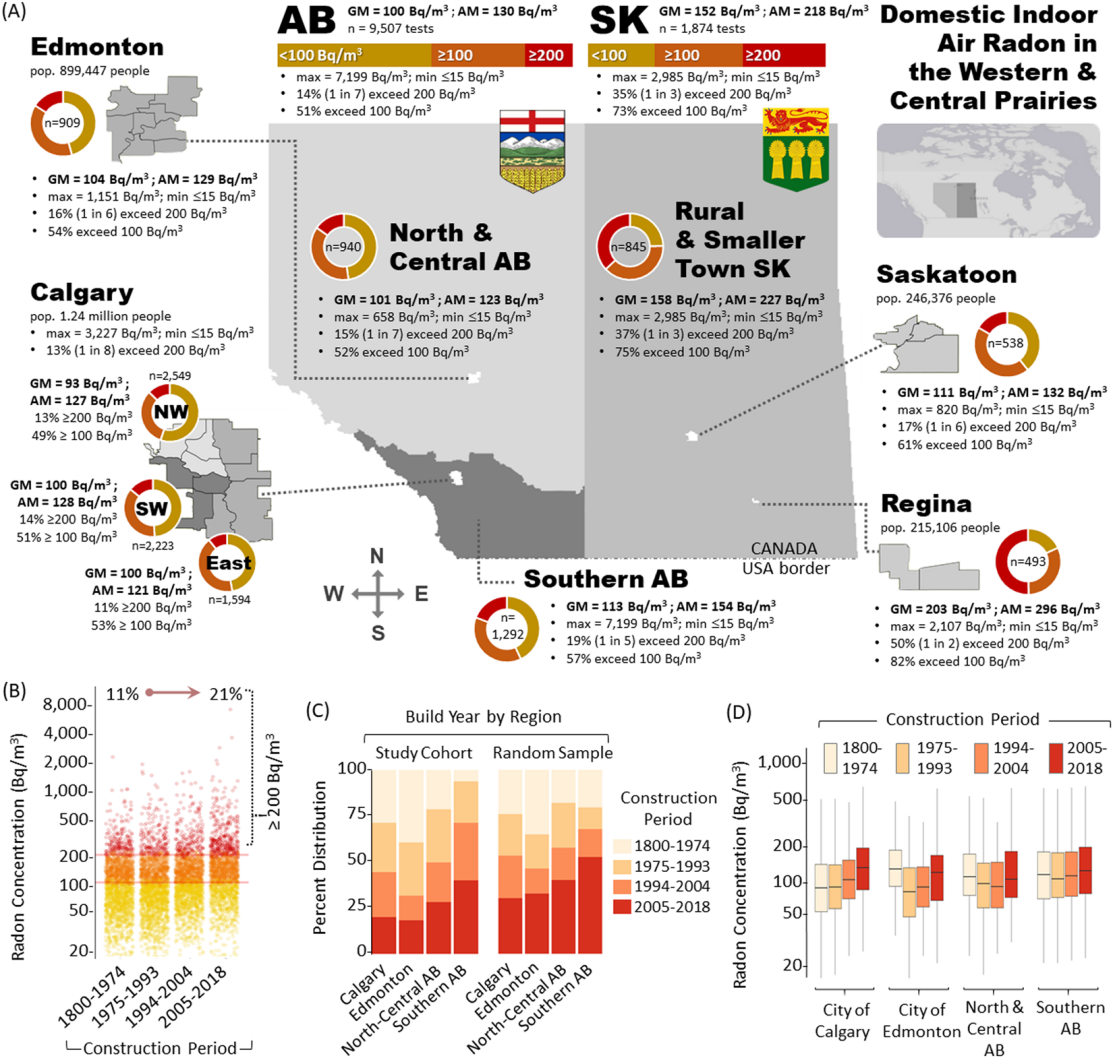
Western Prairie Radon Map and Radon as a Function of Year of Building Construction. Panel A: Administrative map of Western North American Prairies, indicating municipal populations, radon levels (geometric mean, arithmetic mean, min, max, number of tests) and percentage of buildings ≥either 100 or 200 Bq/m^3^ radon. Bar and pie charts show distribution of radon concentration readings for each region, with yellow = 0–99 Bq/m^3^; orange = 100–199 Bq/m^3^; red ≥ 200 Bq/m^3^. Panel B: Domestic indoor air radon concentrations for buildings split into quantiles for year of construction. Yellow dots = 0–99 Bq/m^3^; Orange dots = 100–199 Bq/m^3^; Red dots ≥ 200 Bq/m^3^. All dots are 50% transparent to indicate data densities. Percentages indicate proportion ≥200 Bq/m^3^ over time. Panel C: Using the quantile divisions of construction period as in (**B**), year of construction data distribution by region for either the study cohort (left) or a random sampling of buildings obtained from MLS real estate database (right). Colours are indicated in legend. Panel D: Using the quantile divisions of construction period as in (**B**), domestic radon concentrations (with 95% confidence intervals) divided by region. Colours are indicated in legend.

Our dataset reflected the population-weighed densities of the survey region (5.2 million people in 2016; 79% in AB and 21% in SK), with 83.5% (9,507) of geographically-linked radon readings being from AB and 16.5% (1,874) from SK (Fig. 3A). We used Canadian federal electoral divisions (ED) as standardized, unbiased geospatial units for analysis as they are assembled in a geographically clustered manner free of political gerrymandering^29^, with ~70,000 people in ~28,000 households per ED in SK (ED_SK_) and ~110,000 people in ~42,000 households per ED in AB (ED_AB_). 32/34 of ED_AB_ and 13/14 of ED_SK_ had ≥30 reported tests, with an average of 237 results per ED (Fig. 3A, Table 1). The most precise geographic metric we report is the Forward Sortation Area (FSA, also known as ‘postcode’), enabling us to cluster results by commonly understood regional units (Fig. 3B). To determine the minimum data points needed to reach data equilibrium within an FSA, we calculated confidence intervals at different sample sizes, randomly selecting 5–2000 tests and running simulations 10,000 times (Fig. 3C). Equilibrium was achieved at ≥500 tests (~1% households) per ED_AB_. The geometric mean radon level for AB was 100 Bq/m^3^ (min = <15 Bq/m^3^; max = 7,199 Bq/m^3^), whilst SK buildings contained a significantly (p < 0.0001) higher 152 Bq/m^3^ (min = <15 Bq/m^3^; max = 2,985 Bq/m^3^) (Fig. 4A). A substantial percentage of buildings exceeded national maximum tolerated exposure limits (200 Bq/m^3^), with 14% (1 in 7) AB buildings and 35% (1 in 3) SK buildings considered at serious risk. At the individual ED resolution, some 133 pairwise post hoc Holms-Bonferroni corrected Tukey tests of mean radon levels resulted in p < 0.0005; of these pair tests, 66 included one of the two Regina EDs highlighting this as a regional radon ‘hotspot’.

**Table 1:** Radon results for each federal electoral division.

	ALBERTA Federal Electoral Division	Geo. Mean	Arith. Mean	Min, Max	# tests
	Banff-Airdrie (incl. Canmore, Cochrane, Crossfield…)	110	146	15, 2116	622
	Battle River-Crowfoot (incl. Camrose…)	125	143	21, 323	65
	Bow River (incl. Strathmore, Vulcan, Brooks…)	100	120	18, 386	187
	Calgary Centre	70	89	15, 962	545
	Calgary Confederation	82	118	15, 3227	1016
	Calgary Forest Lawn	90	116	15, 689	126
	Calgary Heritage	102	121	15, 1040	700
	Calgary Midnapore	104	125	17, 1014	716
	Calgary Nose Hill	107	146	15, 1575	585
	Calgary Rocky Ridge	97	125	15, 1513	948
	Calgary Shepard	99	118	15, 1225	615
	Calgary Signal Hill	120	154	15, 1562	978
	Calgary Skyview	97	114	15, 350	137
	Edmonton Centre	114	148	15, 1151	131
	Edmonton Griesbach	126	143	39, 311	38
	Edmonton Manning	68	84	20, 344	44
	Edmonton Mill Woods	95	125	26, 576	44
	Edmonton Riverbend	97	116	22, 551	247
	Edmonton Strathcona	125	152	15, 582	199
	Edmonton West	81	97	15, 374	93
	Edmonton-Wetaskiwin (incl. Leduc…)	87	102	15, 365	90
	Foothills (incl. Okotoks, High River, Pincher Creek…)	124	177	15, 7199	483
	Fort McMurray-Cold Lake (incl. Lac la Biche…)	81	95	16, 269	27
	Grande Prairie-Mackenzie (incl. High Level, Hay Lake)	71	91	23, 576	38
	Lakeland (incl. Vermillion, Vegreville, Athabasca…)	107	134	37, 479	38
	Lethbridge (incl. Coaldale, Picture Butte, Coalhurst…)	97	114	15, 308	102
	Medicine Hat-Cardston-Warner	89	111	17, 501	82
	Peace River-Westlock (incl. Valleyview, Loon Lake…)	114	141	22, 418	26
	Red Deer-Mountain View (incl. Eckville, Bentley…)	114	140	15, 617	106
	Red Deer-Lacombe (incl. Carstairs, Didsbury, Sundre…)	112	145	15, 658	73
	St. Albert-Edmonton	101	134	15, 394	113
	Sherwood Park-Fort Saskatchewan	113	121	26, 651	178
	Sturgeon River-Parkland (incl. Morinville, Redwater…)	101	120	15, 374	60
	Yellowhead (incl. Jasper, Grand Cache, Edson…)	112	142	24, 411	55
	**SASKATCHEWAN Federal Electoral Division**	**Geo. Mean**	**Arith. Mean**	**Min, Max**	**# tests**
	Battlefords-Lloydminster (incl. Moosomin…)	169	226	30, 1271	55
	Carlton Trail-Eagle Creek (incl. Muskeg Lake, Humboldt…)	117	166	15, 1394	100
	Cypress Hills-Grasslands (incl. Swift Current…)	205	290	15, 2985	323
	Desnethé-Missinippi-Churchill River (incl. Big River…)	110	138	20, 350	14
	Moose Jaw-Lake Centre-Lanigan (incl. Whitecap…)	138	181	15, 774	70
	Prince Albert (incl. Wahpaton, Tisdale, Nipawin…)	84	116	15, 840	61
	Regina-Lewvan	214	309	15, 2107	262
	Regina-Qu’Appelle (incl. Lipton, Ituna, Abernethy…)	169	239	20, 1430	121
	Regina-Wascana	191	282	15, 1930	229
	Saskatoon West	109	130	15, 400	107
	Saskatoon-Grasswood	111	130	15, 440	211
	Saskatoon-University	111	136	15, 820	220
	Souris-Moose Mountain (incl. Estevan, Weyburn…)	155	198	20, 860	49
	Yorkton-Melville (incl. Hudson Bay, Yellowquill…)	128	167	15, 640	52

### Household radon concentrations as a function of year of building construction

Significant increases in radon we observed over time when buildings are grouped into year-of-construction quantiles (1800–1974, 1975–1993, 1994–2004 and 2005–2018) (Fig. 4B). Regression analysis indicates a progressive and significant (F (1, 7791 = 235.2, p < 0.0001)) increase in radon with increasing modernity (Supplementary Fig. 2A). The % buildings ≥200 Bq/m^3^ rises from 11 to 21% between the first and last quantile, whilst radon increases (by 40%) from 115 Bq/m^3^ in the early-to-mid-20^th^ century to 161 Bq/m^3^ in 21^st^ century. The distribution of building ages within the cohort were broadly comparable to independently documented norms for the region (reported via real estate multiple listing services (MLS)) (Fig. 4C). By separating quantiles by region, radon levels decreased in North and Central Alberta buildings built from 1975–1993 compared to those built ≤1974 (Fig. 4D). This was not true for Southern Alberta, where 20^th^ century radon remained steady until the 1990s. In all regions, all build types constructed >1990s display an increasingly steep indoor air radon increase.

### Radon as a function of structural attributes of the building

**Figure 5:**
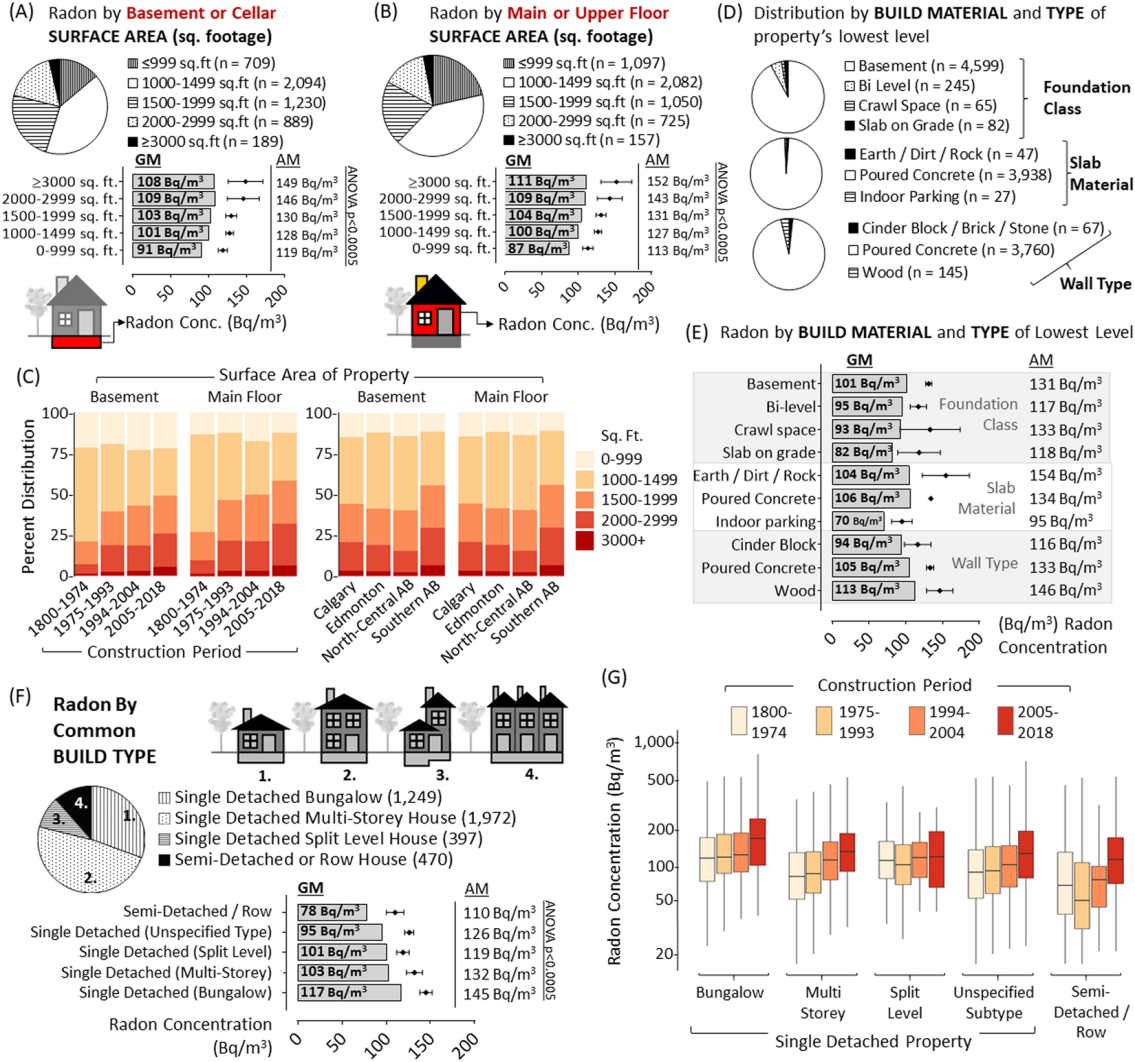
Radon as a function of structural attributes of the building. Panel A: Pie chart shows distribution of reporting. Graph shows geometric mean radon (grey bars) and arithmetic mean radon with 95% confidence intervals (black diamonds with bars) by reported surface area of basement or cellar level of building (in square feet). Panel B: Pie chart shows distribution of reporting. Graph shows geometric mean radon (grey bars) and arithmetic mean radon with 95% confidence intervals (black diamonds with bars) by reported surface area of main or upper level of building (in square feet). Panel C: Surface area of building data distribution for basement or main/upper floor (as indicated), using the quantile divisions of construction period and regional divisions as in Fig. 3B,C. Colours are indicated in legend. Panel D: Pie chart shows distribution of reporting metrics pertaining to the building materials of and general type of the buildings lowest level. Panel E: Graph shows geometric mean radon (grey bars) and arithmetic mean radon with 95% confidence intervals (black diamonds with bars) by reported foundation class, slab material or wall type for lowest level of the building. Panel F: Cartoon indicates the four classes of build type in the study cohort. Pie chart shows distribution of reporting. Graph shows geometric mean radon (grey bars) and arithmetic mean radon with 95% confidence intervals (black diamonds with bars) by reported building type. Panel G: Using the quantile divisions of construction period as in Fig. 3B, domestic radon concentrations (with 95% confidence intervals) divided by building type. Colours are indicated in legend. ANOVA analysis outcomes are indicated on all graphs.

**Figure 6:**
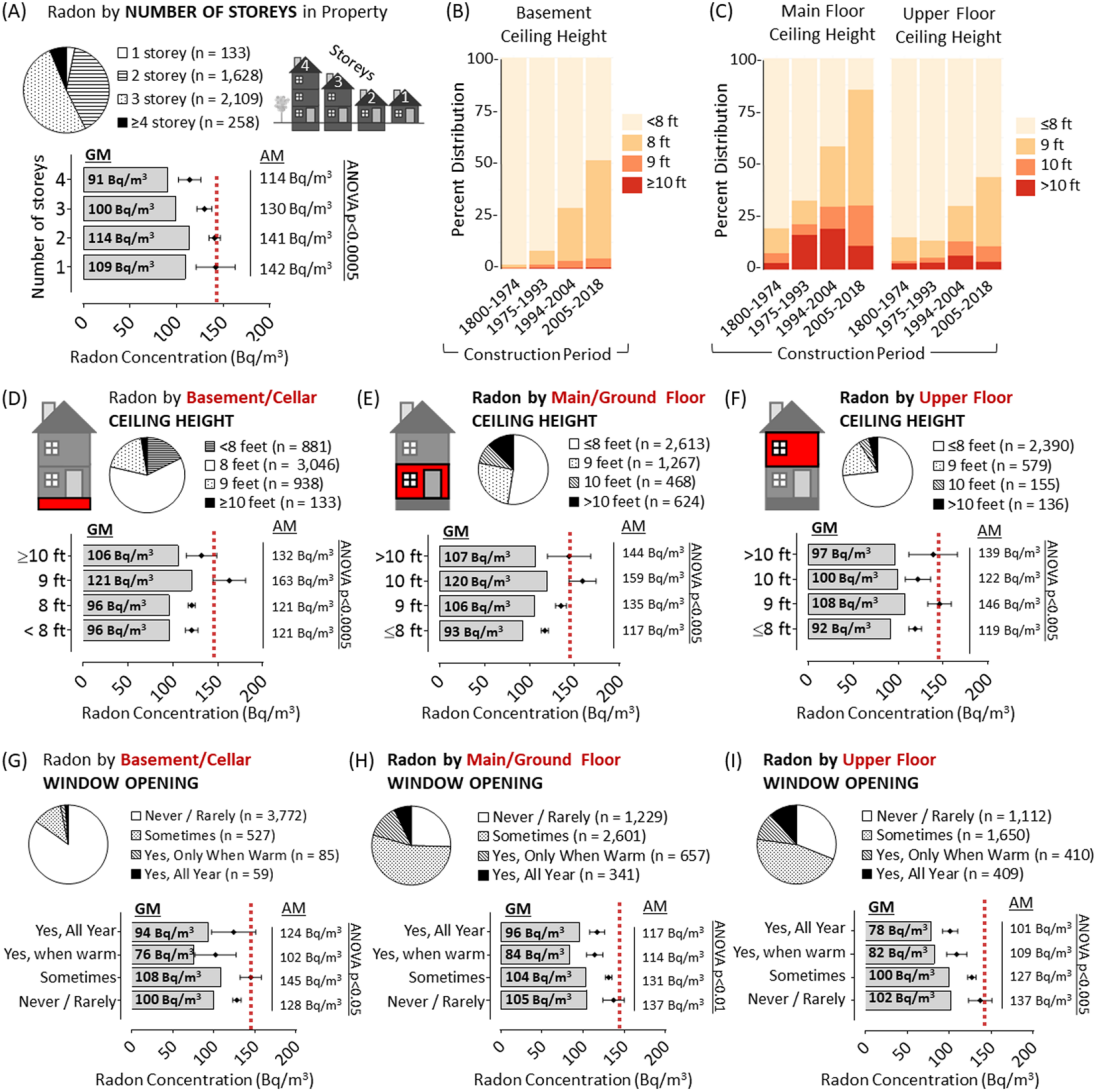
Radon as a function of ceiling height and occupant behaviour influencing building air dynamics Panel A: Pie chart shows distribution of reporting. Graph shows geometric mean radon (grey bars) and arithmetic mean radon with 95% confidence intervals (black diamonds with bars) by reported number of storeys for the building (as indicated by the cartoon). Panel B: Using the quantile divisions of construction period as in Fig. 4B, data distribution for basement ceiling height. Colours are indicated in legend. Panel C: Using the quantile divisions of construction period as in Fig. 3B, data distribution for main floor (left) and upper floor (right) ceiling height. Colours are indicated in legend. Panels D-F: Pie charts show distribution of reporting. Graph shows geometric mean radon (grey bars) and arithmetic mean radon with 95% confidence intervals (black diamonds with bars) by ceiling height (in feet) reported for either basement/cellar (**D**), main floor (**E**) or upper floor (**F**). Panel G-I: Pie charts show distribution of reporting. G Graph shows geometric mean radon (grey bars) and arithmetic mean radon with 95% confidence intervals (black diamonds with bars) by window opening behaviour reported for either basement/cellar (**G**), main floor (**H**) or upper floor (**I**). ANOVA analysis outcomes are indicated on all graphs. Red dotted lines are a reference point of arithmetic mean radon for cohort.

To interrogate changing architectural trends, we analyzed radon by a variety of structural features. There were statistically significant (p < 0.005) increases in radon with larger basement square footage, reported as it would be defined for a formal MLS used in real estate transactions (Fig. 5A). This was also true for the surface area of main/upper level (p < 0.0005) (Fig. 5B). Importantly, analysis indicated that more modern buildings did have increasingly larger surface areas, irrespective of region (Fig. 5C). We next determined whether radon could be distinguished between four regionally-common foundation slab and wall types (see methods for details). Radon did not vary significantly with any of these variables (Fig. 5D,E), although, as most buildings within the cohort had basement type foundations (90%) with concrete walls (95%) and slabs (98%), there is limited power to assess this. Radon was also not significantly altered by the presence (n = 3,594; Geometric Mean = 104 Bq/m^3^) or absence (n = 394; Geometric Mean = 103 Bq/m^3^) of basement plumbing, which are noteworthy ground penetrations in most buildings. Radon levels were also not significantly impacted by the presence (n = 1,030; Geometric Mean = 100 Bq/m^3^) or absence (n = 4,049; Geometric Mean = 101 Bq/m^3^) of a ‘walkout basement’ (i.e. rear-facing basement walls with functional doors open to a recessed ground level), a noteworthy building envelope penetration common in the region. Variance analysis showed significant differences (F (7, 5011 = 5.15, p < 0.0005)) between North American building design types, with single detached bungalows (i.e. a building with a single level above ground) containing the highest radon and a semi-detached or row building containing the lowest (Fig. 5F). This trend was observable irrespective of year of construction, although radon levels still increased with modernity regardless of design type (Fig. 5G). In addition to square footage, we speculated that the greater radon observed in bungalow-type buildings could be a function of total height, which is influenced by the number of storeys (opportunity for dilution) as well as ceiling height of each floor. Indeed, radon was also significantly (p < 0.05) higher in buildings with fewer reported storeys, regardless of specific design type (Fig. 6A). More modern buildings also had taller ceilings, with the majority built in 21^st^ century possessing >9–10 ft ceilings – a rarity for those constructed through most of the 20^th^ century (Fig. 6B,C). ANOVA analysis revealed significant increases in mean radon with increased ceiling height in either the basement/cellar (p < 0.0005), main/ground floor (p < 0.0005) or upper floors (p < 0.005) (Fig. 6D–F). A multiple regression analysis predicted higher radon concentration in buildings with both taller basement and main level ceilings (F (3, 4968 = 51.59, p < 0.0005)).

### Radon as a function of occupant behaviour influencing building air dynamics

As thermal stacking (hot air rising) can promote negative pressure differentials that influence radon entry, we asked participants to indicate household thermostat settings. We observed no significant difference in domestic radon based on occupant thermostat behaviour at any time of day, despite thermostat behaviour differing considerably by time and occupancy status (Supplementary Fig. 2B–E). Building air dynamics, including pressure differentials and dilution effects, may also be altered by window opening behaviours. Whilst basement windows were generally not opened, main floor windows and upper floor windows were opened sometimes-to-frequently (Fig. 6G–I). We observed significant (p < 0.05–0.0005) differences between the reported frequency groups on all levels of the building, indicating that radon levels vary with window opening behaviour. The greatest reductions in domestic radon were where the occupants opened upper floor windows frequently.

### Modern radon seasonal variation

**Figure 7:**
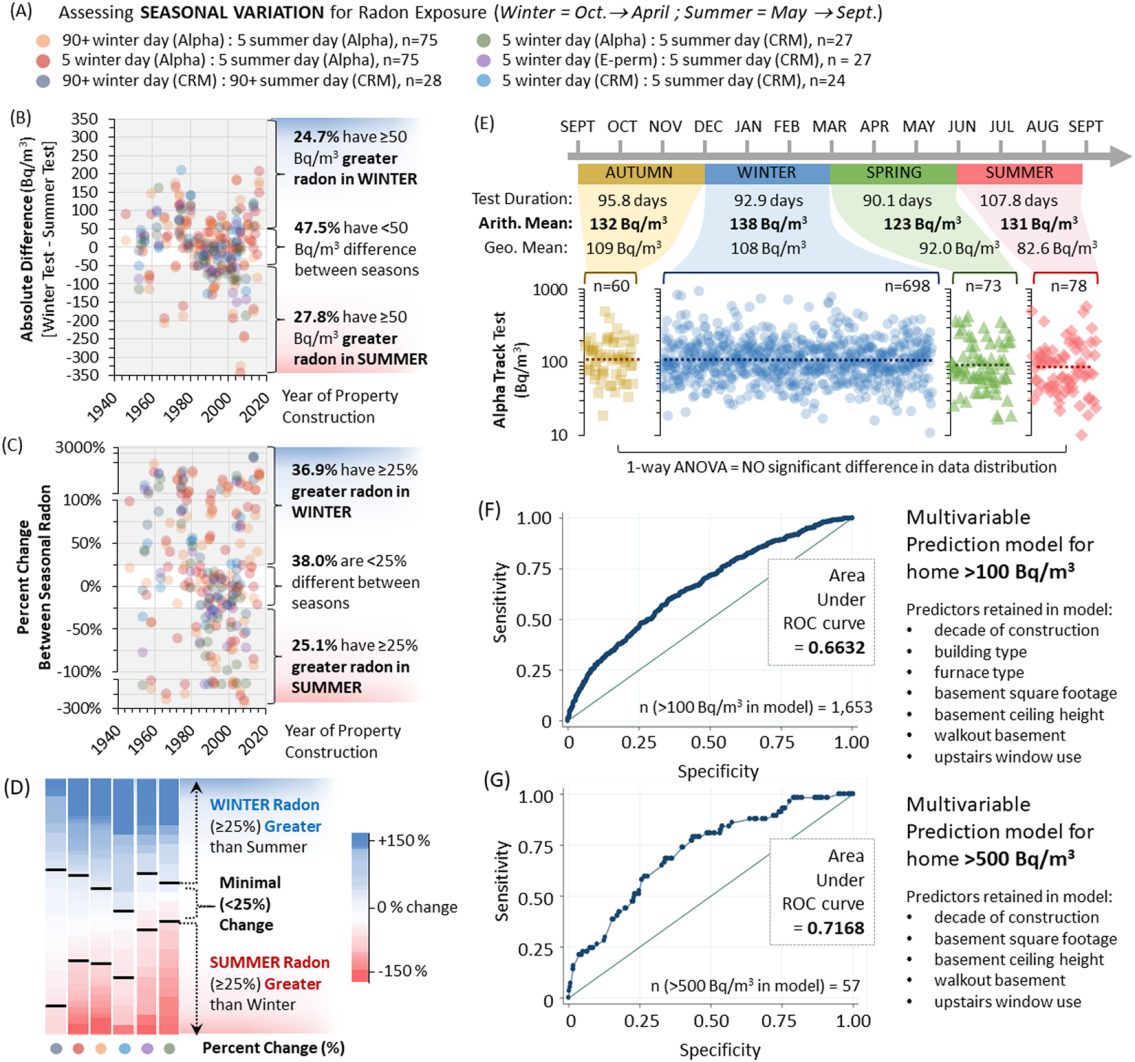
Seasonal variations in radon test outcome and multivariate model. Panel A: All radon testing technologies and test duration permutations used in our study where winter (defined as between October to April) AND summer (defined as May to September) readings were available were paired and colour coded as indicated. Panel B: Using the matched seasonal data pairs defined in (**A**), the absolute difference between a winter radon test result minus the value summer radon test result were calculated, plotting data out by year of building construction. Percentages indicate those with <50 Bq/m^3^ difference between seasons, ≥50 Bq/m^3^ in winter (positive values), or ≥50 Bq/m^3^ in summer (negative values). Panel C: Using the matched seasonal data pairs defined in (**A**), the percent change between a winter radon test and summer radon test result were calculated, plotting data out by year of building construction. Percentages indicate those with <25% overall difference between seasons, ≥25% more radon winter (positive values), or ≥25% greater radon in summer (negative values). Panel D: Heat map analysis of data in (**C**), with values denoted by colours indicated by the legend. Increasing red denotes higher summer radon, increasing blue denotes higher winter radon increasing white denotes lack of change across seasons. Panel E: 90 + day alpha track radon test results were split by season, where the majority (90%) of the 90 + day test window corresponds to one of four seasonal periods defined as: autumn (yellow: Sept, Oct, Nov), winter (blue: Dec, Jan, Feb), spring (green: March, April, May) or summer (red: June, July, Aug). Test duration as well as arithmetic and geometric mean radon levels for all tests in each period are indicated. 1-way ANOVA analysis reveals no statistically significant difference in data distribution or overall radon between these four long term test periods. Panels F-G: Receiver Operator Characteristic Curves for models examining ≥100 Bq/m^3^ and ≥500 Bq/m^3^, as indicated.

To delineate seasonal variations in modern radon exposure, we compared pairs of results obtained from all radon test modalities (placed < 10 cm apart, same building) used in this study, including data from a cohort of 28 buildings that deployed digital pulsed ion chamber continuous radon monitors (CRM) and passive electret ion chambers (E-perm). Paired 5 day from any test combination showed reasonable precision, with r^2^ ≈ 0.80 (Supplementary Fig. 3A–C). For simplicity, we clustered cold month (October-April) readings as ‘winter’ (i.e. heating season) and warmer month (May-September) readings as ‘summer’ (i.e. cooling season), and measured the absolute differences (winter minus summer reading). Combining all pairwise test outcomes (Fig. 7A), 47.5% of buildings showed a minimal (<50 Bq/m^3^) difference, with 24.7% displaying ≥50 Bq/m^3^ greater radon in winter, and 27.8% displaying ≥50 Bq/m^3^ greater radon in summer (Fig. 7B). As monitoring absolute differences may amplify trends in buildings with exceptionally high radon, we also calculated percent difference between seasons, where 0% indicated no seasonal change, positive % indicate increasing winter radon and negative % indicate increasing summer radon (Fig. 7C). Using this, 36.9% of buildings showed greater (≥25%) winter radon, 38% of buildings showed minimal (<25%) change, and 25.1% showed greater (≥25%) summer radon. Heat map analysis confirmed findings across test modalities, also independent of construction year (Fig. 7D, Supplementary Fig. 3D). Using the 90 + day alpha track test cohort, we examined data distribution across a calendar year split into equal, three month periods (autumn, winter, spring and summer) within which at least 90% of the duration of a given radon test took place. In order to control for regional variations, only 90–107 day AB tests were examined. There was no statistically significant difference in the distribution of radon readings across any season (Fig. 7E). Arithmetic and geometric means between seasons were comparable, with only a 7–25 Bq/m^3^ (5–23.5%) difference from winter to summer, and less (0–16 Bq/m^3^; 0–15%) when comparing winter to spring/autumn. This indicates that, for long term (90 + day) tests, season has a relatively minor impact on modern North American radon exposure, and that large datasets collected outside of winter months do not require any significant correction to extrapolate annual dosimetry of populations.

### Overall and subset regression models

When combining all variables to define a maximally predictive set of exposure variables, we started with predictors of buildings ≥100 Bq/m^3^ as there was the largest number of buildings (with complete metrics, n = 1,659) that met this outcome for a stable statistical model. All relevant variables were included in a full model for backwards stepwise elimination, with a p-value threshold of 0.25. All remaining variables were then included in a logistic regression model. Given the relatively small number of homes with levels >500 Bq/m3 we performed these analyses as exploratory given the potential for overfitting our data. Complete modeling results are included in Table 2. We included only the buildings from the Alberta dataset, where near complete measures were available. Interestingly, at least one variable from each group of exposure measure (building characteristics, basement characteristics, building heating characteristics and resident behaviours) remained relevant in our prediction model for a ≥100 Bq/m^3^ building following a backwards elimination approach. The strongest predictors among building and basement characteristics for ≥100 Bq/m^3^ buildings were being a newer, single-detached building with higher basement ceilings, a large basement square footage and a basement with no walkout (Fig. 7F,G). Having a natural gas furnace was generally predictive of buildings having levels ≥100 Bq/m^3^, although the proportions of individuals with other heating types was much lower (<10% non-natural gas buildings). Of all the human behaviour characteristics, the only that remained in the multivariable predictor was the use of upstairs windows, with frequent use being associated with lower likelihood of having ≥100 Bq/m^3^. For prediction models for ≥200 Bq/m^3^, building and heating type were dropped due to a lack of variability in the exposure levels given the smaller numbers of buildings (n = 426) (Fig. 7F). These effect estimates were still consistent in the ≥500 Bq/m^3^ bracket, although the statistical significance of the variables no longer remained due to reduced sample size (n = 57) (Fig. 7G).

**Table 2:** Multivariate Prediction Models.

# homes > level in model	Radon > 100 Bq/m^3^			Radon > 200 Bq/m^3^			Radon > 500 Bq/m^3^		
	1659			426			57		
Decade of Construction	OR	L95% CI	U95% CI	OR	L95% CI	U95% CI	OR	L95% CI	U95% CI
Pre 1960	1.00	Reference		1.00	Reference		1.00	Reference	
1960–1980	1.74	1.29	2.35	1.67	0.96	2.88	0.47	0.07	3.39
1980–2000	1.58	1.19	2.09	1.57	0.93	2.65	1.65	0.37	7.46
Post 2000	3.07	2.31	4.08	2.68	1.61	4.47	3.34	0.77	14.41
**Basement Ft**^**2**^									
<750	1.00	Reference		1.00	Reference		1.00	Reference	
750- < 1750	1.01	0.85	1.21	1.10	0.85	1.43	1.15	0.56	2.37
1750- < 3500	1.06	0.84	1.33	1.14	0.82	1.59	1.64	0.72	3.71
≥3500	1.05	0.69	1.59	1.58	0.93	2.68	2.77	0.91	8.45
**Building type category**									
Semi-detached	1.00	Reference							
Detached	2.13	1.65	2.74						
Other	0.38	0.08	1.78						
**Walkout basement**									
No	1.00	Reference		1.00	Reference		1.00	Reference	
Yes	0.77	0.65	0.93	0.62	0.47	0.82	0.60	0.29	1.22
**Upstairs Window Use**									
Rarely	1.00	Reference		1.00	Reference		1.00	Reference	
Sometimes	1.01	0.86	1.19	1.00	0.80	1.26	0.60	0.33	1.07
Frequently (warm)	0.72	0.57	0.92	0.59	0.39	0.90	0.67	0.25	1.81
Frequently (always)	0.57	0.44	0.72	0.63	0.42	0.94	0.54	0.18	1.58
**Basement Ceiling Height**									
8 ft of less	1.00	Reference		1.00	Reference		1.00	Reference	
9 ft	1.26	1.04	1.54	1.54	1.19	2.00	1.83	1.01	3.32
10 ft or greater	1.38	0.87	2.20	1.82	1.04	3.18	0.65	0.09	4.96
**Furnace/Heat Type**									
Natural gas	1.00	Reference							
Propane or Oil	0.29	0.06	1.50						
Wood	1.07	0.71	1.60						
Electric	0.93	0.64	1.36						

## Discussion

These results reveal a troubling, progressive increase in radon exposure within the modern residential environment of North America. Our previous study of 2,385 greater Calgary area buildings showed a 31.5% increase in radon levels in those constructed since 1992 versus older buildings^15^, although it was unclear whether this trend applied more broadly across North America. This study establishes that the relative ‘modernity’ of the residential environment strongly impacts radon exposure across a broad North American region, with newer homes containing greater and greater radon, and identifies multiple building metric and behavioural variables that associate with this phenomenon. This highlights a highly undesirable and noticeably opposite situation to European countries (e.g. Nordic nations and NW Spain), wherein newer homes display reduced radon relative to older counterparts^30,31^. This begs the question – what are North Americans doing so wrong (or what have Europeans done so right)?

In addition to year of construction, North American design factors that correlate with increased radon exposure include: larger building surface areas (i.e. ‘square footage’, footprint of the building in contact with bedrock and/or soil), taller main/ground floor ceilings, fewer storeys (i.e. bungalow design), basement penetrations, and reduced window opening behaviour on upper levels. The presence of basement level plumbing (active or roughed in), thermostat settings, foundation type or materials, or basement wall types do not significantly correlate with variable radon exposure (please see **Supplementary Information** for further discussion). One interesting factor when considering Western North American residential building stocks is that they display striking uniformity. Indeed, most homes are <100 years old, foundation and construction materials are highly uniform, and build type options are limited. This is in striking contrast to Europe, whose housing stock dates back centuries and is highly diverse in style and construction materials. This North American homogeneity may be viewed as an advantage (it is easier to understand, given smaller variable size) and disadvantage (prevalent factors contributing to high radon are more impactful across populations).

The environmental design metrics we characterized represent a strong starting point for ‘phenotyping’ buildings with features linked to higher or lower radon. In future, it will be important to capture data on air tightness, a variable impacted by the overall number of building envelope penetrations, window glazing thickness and age, as well as roofing type, roofing insulation and insulation thicknesses. These factors are generally different in modern versus older buildings, and many (such as increasing air tightness) are being rapidly modified in housing stocks as part of increasingly vital energy efficiency measures and has been predicted to increase potential future radon-attributable lung cancer risk estimates^32^. We stress, however, that if the air dynamics of a building is balanced correctly, then energy efficiency measures need not contribute to higher radon, and our observations should NOT be used as an argument against energy use reduction strategies.

We also find that existing dogma – that radon is typically higher during winter heating months – no longer applies uniformly, at least within North America, where nearly half of residences showed consistent radon all year, and a quarter demonstrate higher summer radon. There are precedents in other regions for higher summer radon^33,34^. We speculate that this trend may be another consequence of the rising air-tightness of buildings in the region, but that this is also coupled with the growing prevalence of air conditioning units. Indeed, air conditioning prevalence rose by 5–7% in our survey region between 2013–2017^35^. Collectively, this creates indoor air that is less diluted in summer by outside air compared to 20^th^ century norms. Alternatively, and as suggested for higher summer (versus winter) radon documented in the USA state of Alabama, this might also be driven by geological mechanisms^34^.

Our observation that radon data distribution no longer fluctuates substantially across seasons counters current dogma, including the foundation principle of ‘seasonal correction values’ that are widely applied in epidemiological studies for estimating population-level radon exposure^36–38^. At the whole population level, the largest fraction of buildings have minimal seasonal differences, and the remainder (with higher summer or higher winter radon) essentially ‘cancel one another out’. This point is critical for future risk estimations as, based on our results, some recent studies^38^ are almost certainly underestimating the cancer burden attributable to radon by using correction values where none are needed. When projecting future cancer burden estimations, we suggest it is also imperative to account for changing exposure patterns with time, and to base estimations on highly quality controlled datasets that are evenly distributed by build type, floor of testing and with sufficient statistical power within a region to reach data saturation. We observed that data equilibrium at ≥500 tests per ED_AB_ (~1% of households per ED), indicating an ideal percentage of buildings to sample, beyond which further data is increasingly superfluous. Thus, we recommend that sampling 1% of properties in a given region of interest should be the ‘goal’ in future studies of this type. Further, this study supports the notion that recommended seasonal windows for long term radon testing (currently October to April for many countries) could be re-examined, and potentially expanded to a September to May paradigm with little-to-no significant loss of data quality. If given a longer officially recommended window of time to radon test, one anticipated benefit could be to increase net levels of radon testing in the general public.

Our data would not, however, support the widespread usage of short term radon tests for calculating radon exposure, as such data displays strong seasonal precision variations and has little predictive value for exposure estimation. Our data enables the calculation of thresholds for a short term test that could be predictive of a long term reading being either above or below an administrative reference levels. If predictive, such thresholds are potentially useful during events such as a real estate transaction involving a home inspection, wherein long term testing is not practical due to the limited time frame. Such thresholds may be calculated as a given reference level ± three SD (σ3, a 99.7% confidence interval), given the potential number of transactions involved (Supplementary Fig. 2E,F). However, even applying σ3 to threshold calculations reveals that short term testing has extremely limited predictive utility, particularly in summer, with inconclusive outcomes in 58.7% of cases during winter and 97% of cases during summer (Supplementary Fig. 2G,H). Thus, we conclude that short term testing should, under no circumstances, be used as a basis for long term radon exposure estimates. This has implications to the home inspection and industrial hygiene industries where in some countries, such as the USA, the use of short term radon testing during home inspections is widespread and in others, such as Canada, implementing this practice is currently being debated. Please see **Supplementary Information** for more discussion.

Epidemiological pooling studies confirm a statistically measurable and linear increase in relative lifetime risk of lung cancer at ≥100 Bq/m^3^ chronic radon exposure^1,21,22^. In Canada, 200 Bq/m^3^ is the maximum tolerated chronic exposure dose, with advice being to strive for as low as reasonably achievable^8^. In the USA, 148 Bq/m^3^ (4 pCi/L) is used as an action level. 17.8% (1 in 6) residential buildings in our survey region exceeded 200 Bq/m^3^, and more than half exceeded 100 Bq/m^3^. Analysis indicates that 15–16.6% of all lung cancers in the region are radon-attributable^38,39^, representing a considerable disease and economic burden. It is worth noting that the 2010 Canadian Build Code – adopted variably by Canadian Provinces between 2010 and 2016 – includes the addition of a sub-slab depressurization radon mitigation device ‘rough-in’ to the foundation. Whilst not an active radon reduction measure at build, this should, in theory, make it easier for Canadians to subsequently install mitigation devices. However, our data clearly demonstrates that this strategy has not had a major impact on radon exposure so far, with properties built from 2010–2018 having the highest radon documented to date.

It is important to stress that ALL North American Prairie regions examined contained buildings that exceeded reference thresholds. There were some regional differences, with the city of Regina containing 200–250% greater radon versus other major urban centres. Whilst Regina contained the highest average radon, regional maxima were observed within rural Southern Alberta nearing the USA border, at 7,199 Bq/m^3^. This is the equivalent of a very substantial 337.6 mSv/yr radiation dose – more than 165X normal terrestrial background levels of ionizing radiation (2 mSv/yr). Based on the underlying geologies, climates and highly similar build practices, it is highly likely that these trends apply to north-central USA states (Montana, N. and S. Dakota, Minnesota, Wisconsin, Illinois, Ohio, Iowa, Nebraska, Wyoming, Colorado), as well the Canadian province of Manitoba, the interior of British Columbia, the Greater Toronto Area, the Ottawa capital region, parts of Quebec and all Maritime provinces.

Please see **Supplementary Information** for a comprehensive analysis of the strengths and limitations of this work. This study: (1) outlines best practice for radon testing large populations for optimal precision and accuracy; (2) raises questions as to the utility of seasonal correction values for estimating radon dosimetry; (3) challenges the validity of short term radon test practices from data reliability standpoint; (4) highlights multiple North American regions of substantial and worsening radon exposure that contrasts starkly with the European experience; and, finally, (5) emphasizes the importance of understanding the modern residential environment as a critical – but modifiable – vector controlling human radiation exposure linked to serious disease incidence.

## Methods

### Main study design

All methods were carried out in accordance with protocols approved by the Research Ethics Boards (REBs) indicated below, adhering to regional guidelines and regulations for research involving citizen science participants. The survey region included the Western North American Prairie provinces of Alberta (AB) and Saskatchewan (SK), which as indicated in Fig. 1 is a region of expected high radon potential based on geologic analysis. From 2010–2018, AB and SK residents purchased or were provided alpha track 90 + day radon detectors that they then deployed and returned for analysis, subsequently receiving their specific radon reading in a confidential manner directly from radon testing laboratories. It is important to stress that kits were inexpensive, offered ‘at cost’ and were not considered to represent a significant economic barrier to participation. Further, nearly all residential buildings in the region possess basements or cellars, are constructed using similar foundational materials and, given these similarities, it is very difficult for any resident within the region to reasonably suspect their property may have ‘high’ or ‘low’ radon. Hence, possible selection biases have been considered and controlled to the fullest extent. The majority (n = 8,033) of these citizen scientist participants were enrolled via the University of Calgary-based ‘Evict Radon’ study, which additionally collected environmental design and behavioural information provided by participants. 2,698 tests were generously provided by the Lung Associations of Alberta/NWT and Saskatchewan, coupled to geospatial information (Forward Sortation Area of residents post code, FSA) and testing period information. 515 tests were obtained from a study conducted by the Saskatchewan Health Authority. We stress that no data from any constituent part of our radon testing cohort were from known lung cancer cases. All groups conducted a random recruitment (any who wanted to join) using similar protocols, and pooled our results to increase statistical power. There was no statistical difference in radon outcomes between datasets, other than that documented by geographic region. Participants obtained tests at cost for CAD$45–60 each (variable depending on test year due to inflation), which were then quality controlled and distributed centrally by researchers. Homeowners and renters were equally eligible. Commercial buildings were not considered. The study design, methodology and data sharing between groups was approved by the Conjoint Health Research Ethics Board, Research Services, University of Calgary and conferred Study Ethics ID = REB17–2239. Records of informed consent were obtained in all cases. For a subset of SK tests (n = 283), part of the ‘Radon Home Health Study’ conducted by the Saskatchewan Health Authority (SHA), device costs were paid for by the SHA Provincial Laboratory and results were not returned directly to participants, instead were first provided to researchers who then communicated to participants. This sub-component of this study received ethics approval via the Biomedical Human REB at the University of Saskatchewan (Study Ethics ID = BIO 15–307). Records of informed consent were obtained in all cases.

### Communications and enrollment

Public outreach was achieved through print media, public seminar, online (website and social media) and mass media messaging via organic (unpaid) TV/radio exposure in an untargeted manner. To join, all participants consented to semi-anonymously provide researchers radon data, with the understanding they and their building’s specific postal address would never be publicly identified. Participants were permitted to withdraw at any time. The Evict Radon cohort also consented to collect and provide data on the radon-tested building’s construction year, build type, foundation type, furnace type, heat-delivery type, floor tested, room of deployment, ceiling heights, thermostat settings, window opening behaviour, basement and ground floor surface area (square footage) and thermostat settings. Rigorous care was taken to educate participants in the correct test deployment methods through communication with Canadian National Radon Protection Program (C-NRPP)-certified professionals and close adherence of advice and testing protocols to Health Canada’s guidelines. For long-term (90 + day) alpha track radon tests, participants were advised to place devices on the lowest level of the building occupied for approximately four or more hours per day, for a minimum of three months during the typical Canadian heating season (October to April).

### Foundation, slab and wall classifications

The four regionally common foundation types were: *basement* (sub-surface and habitable level of building), *crawl space* (subsurface level where ceilings are too low to stand up, not habitable), *slab-on-grade* (a mold set into the ground and filled with concrete, leaving no empty space between the ground and the structure) or *bi-level* (basement staggered with slab-on-grade, also called ‘split level’). We also examined by slab material (concrete, indoor parking or compacted dirt/earth/rock), or wall type (wood, concrete or cinder blocks/brick/stone).

### Thermostat setting and window opening classifications

We asked participants to indicate household thermostat settings within typical domestic temperature ranges (<16 °C to >23 °C). Four different periods were considered: (1) daytime with the building unoccupied, (2) daytime with the building occupied, (3) evenings with building occupied and (4) nighttime when occupants were asleep. Participants also indicated whether windows were opened on each building level (basement, main or upper) in four categories: (1) frequently – always (all times of year), (2) frequently – warm (only when it is hot), (3) sometimes and (4) rarely.

### Radon test comparison subsets

The 2017–2018 Evict Radon study cohort was segregated into representative groups encompassing a normalized distribution for build year and location within the region, and then 1,000 buildings were randomly selected for additional deployment of an alpha track 5-day radon detector to be placed side-by-side with the standard 90 + day radon test for the final 5-days of the test period. Of the 704 buildings who successfully deployed and returned 5-day alpha track tests during winter months (March-April 2018), 100 were invited to deploy a second 5-day alpha track test in the same location during the summer (July-August 2018). Participants were advised to use ‘closed-house’ conditions during this summer period, so to reflect the air dynamics of their analogous 5-day winter test as best possible. A subset of 28 buildings, confined within but evenly distributed across a specific geographic region to control for meteorology trends (NW quadrant of the city of Calgary), were also selected for passive electret ion chamber radon device deployment (E-Perm type devices) and digital pulsed ion chamber Continuous Radon Monitors (CRMs).

### Technology specifications, data collection, storage and reporting

A total of 11,727 radon tests were returned and eligible for analysis, representing the aggregation of multiple-year testing efforts by Evict Radon, the Lung Associations and Saskatchewan Health Authority. Alpha track radon tests were closed passive etched track detectors made from CR-39 plastic film inside antistatic and electrically conductive housing with filtered openings to permit gas diffusion, with a typical linear range of 15 to 25,000 Bq/m^3^. To be read, CR-39 films are etched in 5.5 N NaOH at 70 °C for 15.5 min and scored using TrackEtch® software at C-NRPP accredited laboratories. The majority of alpha track radon tests in this study were analyzed by Radonova laboratories, Sweden (ISO17025 certified). 90 + day alpha track tests were Radtrak2 devices, whilst 5-day alpha track tests were Duotrak devices both obtained from and analyzed by Radonova. Electret devices were e-PERM short term radon tests from and analyzed by AGAT laboratories (Canada). Continuous radon monitors were RadonEye + digital pulsed ion chamber devices from FTLab (South Korea). Controls included duplicates to ensure device reproducibility, spiked positives (to ensure accuracy) and non-deployed negative “blanks” (controlling for transport and storage prior to analysis). Readings throughout this study are in Bq/m^3^ rounded to the nearest whole number.

### Geospatial analysis

Readings linked to useable FSA geospatial data (n = 11,402) were grouped into larger federal electoral divisions (ED). ED encompass administrative divisions as defined by the 2013 *Representation Order*, and are a convenient way to group datasets as each ED are population-weighted approximately equally within a given province (Alberta = 107,000 people per ED, Saskatchewan = 74,000 people per ED) (Elections Canada, 2015). Geospatial analysis was run in ArcGIS pro, and maps were produced with ArcGIS pro or Inkscape. Some unique regions comprised of consolidated EDs were used for ease of interpretation, typically fusing multiple districts from the same city. It should be noted that geotagging relies on accurate reporting of FSA, and 325 radon tests (2.8% of total) could not be matched with sufficient certainty to be included in geospatial analysis. To calculate isolation factors, buildings were geolocated using satellite imaging available via Google Maps. From the centre point of all surrounding human structures (or, more rarely, bodies of water) in all directions, lines were measured using distance and area mapping tools to isolate a circumference around the building free of visible human buildings, allowing the calculation of areas (in m^2^). This value was labeled ‘isolation factor’ with an increasing value reflecting a larger area surrounding the building with no neighbouring buildings.

### Geological analysis

Radon potential maps were created with an initial review of the available United States Geological Survey and Environmental Protection Agency radon potential map to help define Canadian-specific methodology^40^. The geological base for the analysis was^27,41^ and the EPA Air and Radiation report (6604 J) 402-R-93–071. The Canadian subset of the geology dataset together with geophysical information from the USGS Mineral Resource Database including the equivalent uranium (eU) concentration in ppm and absorbed dose (total exposure rate at ground level) data were used in the map construction. Uranium geochemical information required levelling as data was incorporated from many regional surveys using different analytical methods. British Columbia data^42^ (representing an existing leveled dataset that was similar to other distributions) were selected as the standard value against which all the other samples were levelled. Geologic map polygons from the levelled datasets were collected in a geographic information system (GIS) format, and the radon potential value of each geology polygon was determined by overlaying the bedrock geology on the USGS Generalized Geologic Radon Potential of the United States and ranking each polygon as a Radon Class Value (RCV) between 1 and 3. The RCV was then used to rank the relative radon potential of all the geology polygons. Geochemical and geophysical datasets provide direct measurements and were given a higher weighting than the extrapolated geology-based radon potential values. Airborne radiometric data from the USGS Mineral Resource Database had the best correlation with radon potential from the analysis of US information, and was given the highest influence in assigning radon potential classes for the Canadian map. Classifications identified high (>300 Bq/kg) and low (<100 Bq/kg) polygons. To be considered high, there was at least one dataset value in the upper quartile of valid polygons, and to be considered low there was at least one dataset value in the lowest 30% of valid polygons, with the remainder (100–300 Bq/kg) encompassing all other points.

### Statistical analysis

Statistical analysis was carried out using R (3.5.1) and RStudio (1.1.456); unreported or internally inconsistent data were excluded. One-way ANOVAs were carried out to test radon levels between groups (building metrics/year of construction/ED), with Bonferroni-Holm post-hoc testing carried out for pairwise comparisons if the ANOVA reached significance. Regressions were run on Ln transformed radon concentrations. In order to define those variables most predictive of having high levels in the building, we conducted a series of multivariable logistic regression models for ≥100 and ≥500 Bq/m^3^. We included all relevant variables in a full model (decade of construction, building type, Basement square footage, foundation type, foundation wall type, slab type, walkout basement, basement ceiling height, heating source, furnace type, presence of plumbing in basement, regular window use (basement, main floor, upper floor, regular exhaust fan use) and performed backwards stepwise elimination with a p-value threshold of 0.25. We then included all of the remaining variables in a logistic regression model and the estimated the area under the receiver operator characteristic curve c-statistic as measure of predictive performance for each radon level. Given the relatively small number of buildings with levels >500 Bq/m^3^ we performed these analyses as exploratory given the potential for overfitting our data.

## Supplementary information

Supplementary Information and Figures

## Data Availability

The de-identified raw data sets generated by the current study are available to academic researchers at public institutions following reasonable request to the corresponding author, and will require a data transfer agreement. Data may not be used for private, commercial, or for-profit purposes for any reason.
