# Supplementary material for: Radon exposure is rising steadily within the modern North American residential environment, and is increasingly uniform across seasons
Source: Sci Rep. 2019 Dec 3;9:18472. doi: 10.1038/s41598-019-54891-8 (PMC6890734; doi:10.1038/s41598-019-54891-8)
Supplement: Supplementary file 1 — Supplementary Information and Figures [file 41598_2019_54891_MOESM1_ESM.pdf]

Radon exposure is rising steadily within the modern North American residential environment, and is increasingly uniform across seasons

Fintan K. T. Stanley[1], Jesse L. Irvine[1], Weston R. Jacques[1], Shilpa R. Salgia[1], Daniel G. Innes[2], Brandy D. Winquist[3], David Torr[4], Darren R. Brenner[5], Aaron A. Goodarzi[1*]

Author Affiliations:

[1]Robson DNA Science Centre, Charbonneau Cancer Institute, Departments of Biochemistry & Molecular Biology and Oncology, Cumming School of Medicine, University of Calgary, Calgary, Alberta, Canada; [2]Radon Environmental Management Corporation, Vancouver, British Columbia, Canada; [3]College of Medicine, Community Health and Epidemiology, University of Saskatchewan, Swift Current, Saskatchewan, Canada; [4]Public Health Physicians of Canada and Saskatchewan Health Authority, Saskatchewan, Canada. [5]Robson DNA Science Centre, Charbonneau Cancer Institute, Departments of Cancer Epidemiology & Prevention Research and Community Health Sciences Cumming School of Medicine, University of Calgary, Calgary, Alberta, Canada.

*Correspondence to: Aaron Goodarzi, A.Goodarzi@ucalgary.ca

Supplementary Discussion – Strengths and Limitations of this Study

This work represents one of the larger residential radon dosimetry studies in North American history and, with a large, highly quality controlled sample size (>11,000 measurements), has analyzed radon test outcome and patterns by region, season, building metrics and occupant behaviours. There are clearly interactions between build type, location and behaviour that allow us to reasonably predict whether a property may or may not >100 Bq/m^3. However, our predictive power is limited for higher dose thresholds, and we conclude that additional data is needed to test and consolidate this. It is possible that our recruitment through public outreach (versus direct contact recruitment) applied some selection bias; however, as this was untargeted and we accepted all valid data any residence within the region, this impact was minimized. Nevertheless, our data set is a convenience sample in contrast (for example) to the Health Canada survey[1] that is a random sample of households that agreed to radon test following random phone survey. However, we should emphasize that we do not demographically restrict any of our outreach or recruitment protocols, and so those opting into our study after randomly encountering our radon awareness material are in many ways approximate to those randomly chosen for study invitation. Indeed, demographic and geographic analysis indicates that there is minial bias within our dataset compared to statistical regional norms. For some home metric data points, we fell short of 100% response rate, but still achieved an average 80-90% response. One advantage to an opt-in mode of radon testing across the region is a very high rate of kit returns and data collection, as participants were highly engaged in the process as citizen scientists. This mode of study is of major interest to modern research, and is now accepted as part of research ethics approval processes[2]. One possible disadvantage is that the minor costs incurred by the participants could have biased our cohort, although again this did not appear to impact building

feature distribution within our study, as compared to regional norms. As such, our sample actually covers the most at-risk and largest section of the population of interest, and represents a major systematic examination of radon with a very large geographic area.

Supplementary Discussion – Building Metrics and Radon

Increased contact between a building and underlying radon-genic materials is a logical driver for increased radon entry, as (i) greater surface area affords greater overall entry points for soil gasses across the foundation and (ii) larger concrete foundational slabs have fixed ratio of shrinkage per surface area whilst curing[3,4], and thus larger slabs have larger gaps where they meet basement walls (enabling greater soil gas entry). Although not a significant predictor in our study, we acknowledge that our statistical power to make conclusions regarding foundation build materials is limited as the majority of buildings in the region (and, indeed North America in general) are made from concrete. That being said, it is known that newer concrete, now often made from recycled aggregate, shrinks more per unit area[5] and, as such, is potentially a contributor to higher radon in more modern buildings. We speculate that taller ceiling heights up to 10 feet may contribute to higher radon by facilitating larger negative pressures at lower levels due to more powerful thermal stacking. Above 10 feet, we suggest that dilution effects due to exceptional internal air volumes within the building likely come into play, partly counteracting thermal stack effects. The influence of thermal stacking is not entirely certain, however, as internal temperatures, at least within a typical domestic range of variation, did not influence radon levels significantly. It is possible that the domestic temperature variations (~7°C), are insufficient to influence stacking. As thermal stacking will also be influenced by roof insulation materials, future work is needed to better understand this. Reduced air exchange dynamics via infrequent window opening is another possible reason for increased radon. Following this same logic, we suggest that one reason that buildings with fewer storeys demonstrate higher overall radon is that they typically have fewer overall air exchange points (such as windows) compared to multi-storey buildings.

Supplementary Discussion - Radon Test Duration Implications to Industry

Whilst major health authorities in Canada, Europe and the USA all officially promote long term testing, many national radon testing and building inspection industries are offering, promoting and performing short term radon tests. Our findings may not entirely preclude the usage of short term tests in specific contexts (e.g. confirming the efficacy of radon mitigation measures that have been undertaken based on a long term reading), we suggest that data from short term tests should not be used for any major cancer risk estimations or health and home decision-making, as there is an unacceptably high risk of false negative or false positive test outcomes relative to national exposure reference thresholds. This would include decisions during real estate transactions

or referral to early cancer detection screening programs. It is worth noting that, based on Canadian Real Estate Foundation data (2008-2018), the majority of real estate transactions in the region occur during warm months (**Supplementary Figure 3D)**. Based on costs (CAD$125-400 per test, source: Canadian Association of Radon Scientist and Technologists), and the relatively high incidence of uncertain outcomes, the widespread use of real-estate transaction based short term radon testing could have a significant economic costs with limited benefit in terms of reliable information gained by consumers (**Supplementary Figure 3E)**. Given this and the limited time frames involved, it is clear that a real estate transaction is an unsuitable scenario for the public to gain reliable radon exposure data for a given building. Radon testing should be performed for 90+ days either before a residential building is listed, or after it is purchased (particularly if renovations are to take place that could alter the air dynamics of the building). This notion is supported by previous studies examining short term testing efficacy in the USA and Europe[6,7]. Hence, the conclusions of this study have implications to the home inspection and industrial hygiene industries where in some countries, such as the USA, the use of short term radon testing during real estate transactions is already widespread and in others, such as Canada, implementing this practice is currently being debated.

Supplementary References

1. Health Canada. *Cross-Canada Survey of Radon Concentrations in Homes - Final Report*, 29 (Government of Canada, Ottawa, Ontario, 2012).
2. Oberle KM, Page SA, Stanley FKT & Goodarzi AA. A reflection on research ethics and citizen science. *Research Ethics* **In Press**(2019).
3. Gilbert RI. Shrinkage, Cracking and Deflection-the Serviceability of Concrete Structures. *Electronic Journal of Structural Engineering* **1**, 2-14 (2001).
4. Videla CC, Carreira DJ, Garner NJ & McDonald DB. Guide for Modeling and Calculating Shrinkage and Creep in Hardened Concrete. Vol. ACI 209.2R-08 (American Concrete Institute, Farmington Hills, MI, 2008).
5. Silva RV, deBrito J & Dhir RK. Prediction of the shrinkage behavior of recycled aggregate concrete: A review. *Construction and Building Materials* **77**, 327-339 (2015).
6. Barros N, Steck DJ & William Field R. Utility of Short-Term Basement Screening Radon Measurements to Predict Year-Long Residential Radon Concentrations on Upper Floors. *Radiat Prot Dosimetry* **171**, 405-413 (2016).
7. Ruano-Ravina A, Castro-Bernardez M, Sande-Meijide M, Vargas A & Barros-Dios JM. Short- versus long-term radon detectors: a comparative study in Galicia, NW Spain. *J Environ Radioact* **99**, 1121-6 (2008).

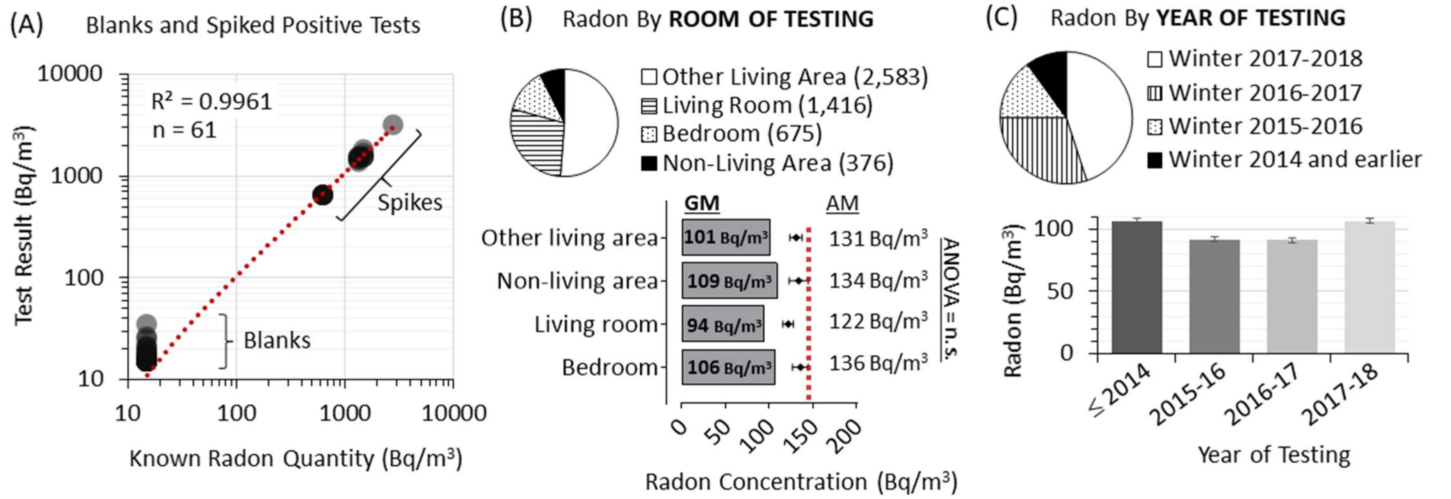

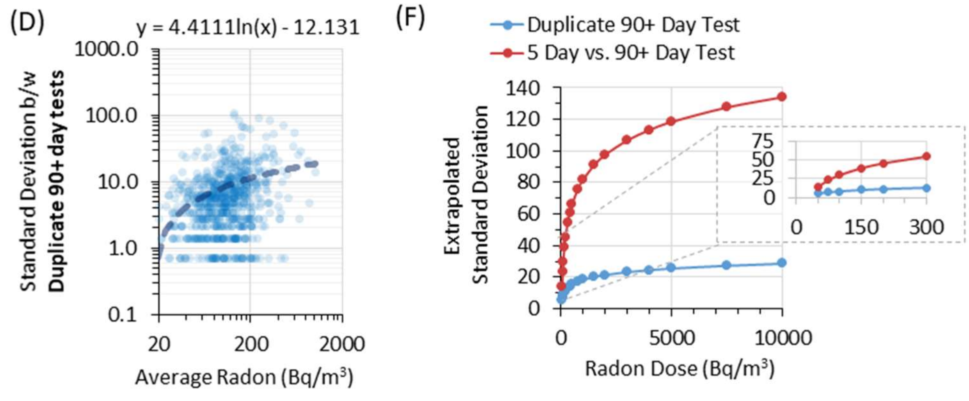

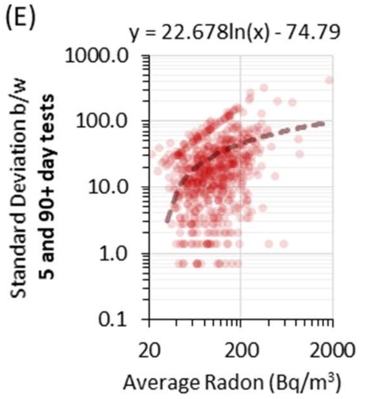

(G)

Standard Deviation Between Test Pairs

Bq/m3	W90xW90	W90xW5*	W90xS5**
50	5.125	5.140	43.504
75	6.913	14.988	59.284
100	8.182	21.975	70.479
150	9.971	31.822	86.259
200	11.240	38.809	97.455
500	15.282	61.063	133.114
1000	18.339	77.898	160.089
2000	21.397	94.732	187.064
5000	25.438	116.986	222.724
10000	28.496	133.821	249.699

* W90xW5 = 90 Day winter test vs. 5 Day test in winter

** W90xS5 = 90 Day winter test vs. 5 Day test in spring-summer

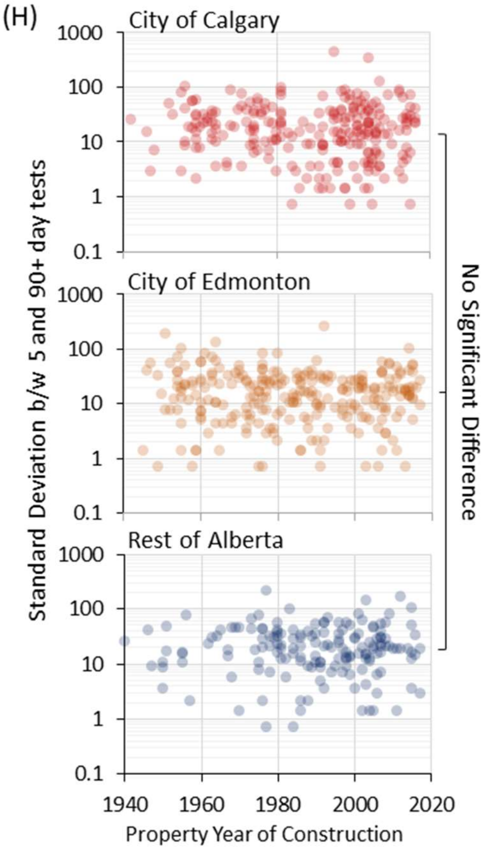

SUPPLEMENTARY FIGURE 1: Radon controls and statistical analysis of short term testing. Panel A: 90+ day alpha track test devices were exposed to known quantities of radon gas, including blanks kept within manufacturer gas-impermeable packaging. As lowest test result sensitivity is a value of <15 Bq/m^3, blanks were marked at 15 Bq/m^3 as the "known quantity". Test results were plotted against known exposures (50% transparent black dots to indicate data density) with linear regression (red dotted lines). Panel B: Pie chart shows distribution of reporting. Graph shows mean radon concentrations (with 95% confidence intervals) by room of test placement. Panel C: Pie chart shows distribution of reporting. Graph shows geometric mean radon concentrations with standard error of the mean (SEM) for each testing year period of our study. Panel C: Raw data showing Ln-transformed radon levels as a function of a building year of construction. Panel D: Standard deviations (SD) calculated for concurrent 90+ day winter alpha track radon tests as a function of average radon between each test. Blue dots are 50% transparent to indicate data density. Dashed blue line indicates logarithmic regression. Panel E: Standard deviations (SD) calculated for a 90+ day winter versus 5 day winter alpha track radon test as a function of average radon between each test. Red dots are 50% transparent to indicate data density. Dashed red line indicates logarithmic regression. Panel F-G: Formulas from (A,B) were used to extrapolate SD across a 10,000 Bq/m3 radon outcome for winter readings. Panel H: Data from Figure 2A was split by testing region outlined in Figure 7A and expressed as a function of year of construction.

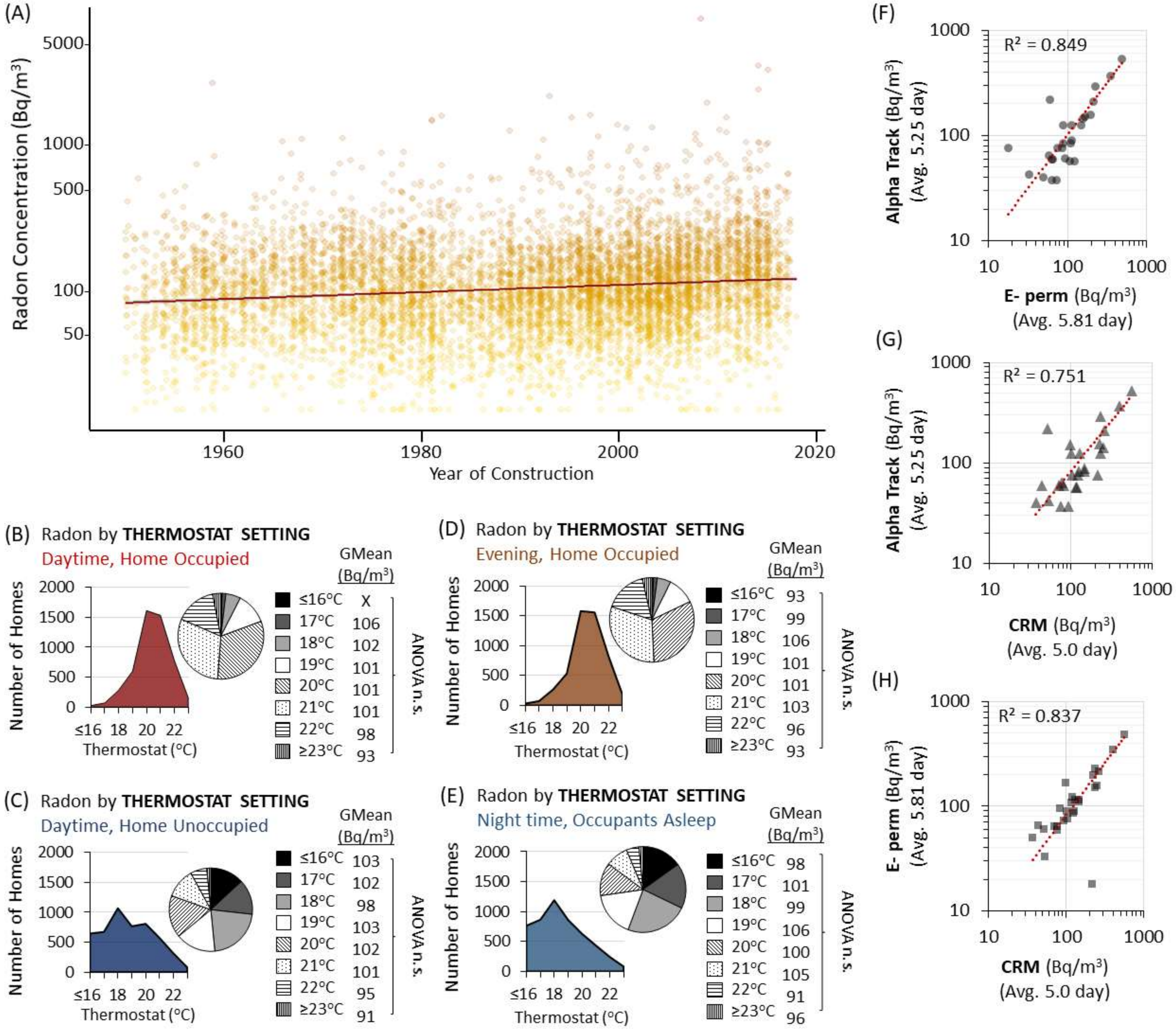

SUPPLEMENTARY FIGURE 2: Radon as a function of build year, thermostat settings and test device technology type. Panel A: Raw data of radon level as a function of building year of construction. Panels B-E: Pie charts and graphs show distribution of reporting for thermostat settings during either daytime with the building occupied (B), daytime with the building unoccupied (C), evening with the building occupied (D) and night time with occupants asleep (E). Mean radon concentrations by thermostat setting are indicated. ANOVA analysis indicates no significant difference between any dataset. Panel F-H: In 28 buildings located in the NW quadrant of the City of Calgary, 5 day alpha track devices were deployed alongside 5 day passive E-perm devices and digital continuous radon monitors (CRM) during winter months. Data points from each test technology modality were plotted against one another (50% black transparent dots, triangles or squares to show data density) with linear regression (dotted red lines).

(A)

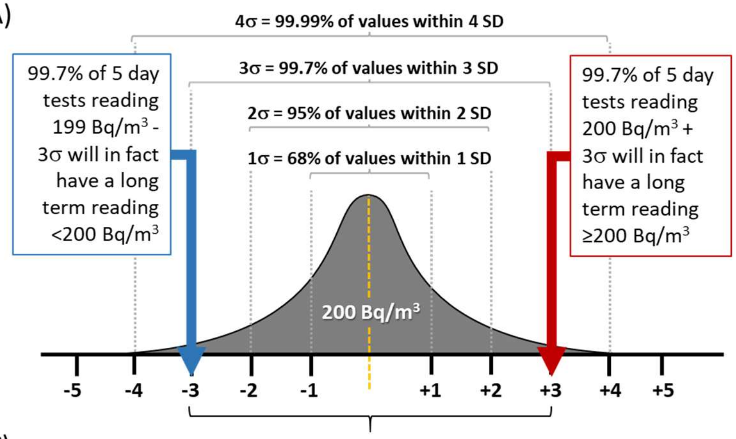

(B)

YEARS:	2008-2018
SALES:	5,213,219
1σ	1,654,212
2σ	237,207
3σ	14,076
4σ	334
5σ	5.2

Based on ~5.2 million Canadian Real Estate Transactions (documented 2008-2018), the projected number of potential short term radon tests falling outside standard deviation (sigma) ranges between 1 to 5. **A 3σ (99.7% confidence interval) is required to keep potential 'erroneous' decisions at <15,000 / decade.**

(C)

COLD MONTH Short Term Test Thresholds (Bq/m³)

	Ref. Level	SD	3σ	LOWER*	UPPER**
WHO	100	21.975	65.924	33.076	165.92
USA	150	31.822	95.467	53.533	245.47
CAN./EU	200	38.809	116.43	82.572	316.43

WARM MONTH Short Term Test Thresholds (Bq/m³)

	Ref. Level	SD	3σ	LOWER*	UPPER**
WHO	100	70.479	211.44	0	311.44
USA	150	86.259	258.78	0	408.78
CAN./EU	200	97.455	292.36	0	492.36

* Lower Threshold = [Reference Level – 1] - 3σ

** Upper Threshold = [Reference Level] + 3σ

(D)

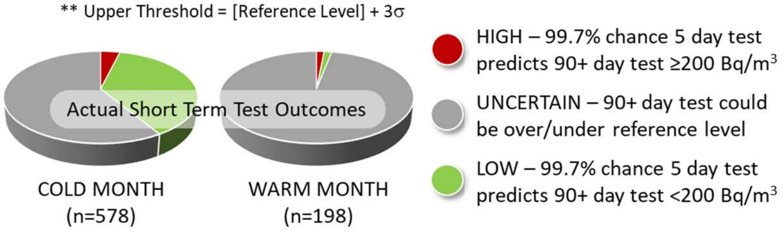

(E)

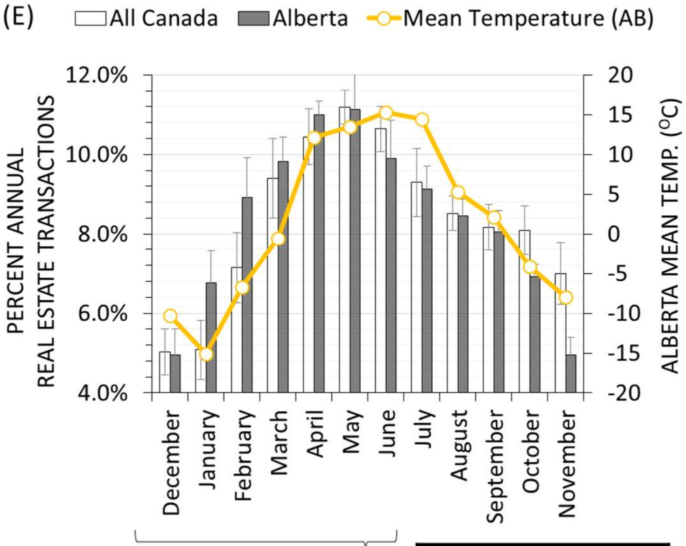

ALL CANADA

	Outcome	%	# / year	Short Term Test Cost COST (CAD$125)	COST (CAD$400)
COLDER MONTHS (Oct-Mar)	High	3.8%	8,003	$ 1,000,435.50	$ 3,201,393.60
	Low	37.5%	78,982	$ 9,872,718.75	$ 31,592,700.00
	Uncertain	58.7%	123,633	$ 15,454,095.75	$ 49,453,106.40
WARMER MONTHS (Apr-Sept)	High	1.5%	5,291	$ 661,434.38	$ 2,116,590.00
	Low	1.5%	5,291	$ 661,434.38	$ 2,116,590.00
	Uncertain	97.0%	342,182	$ 42,772,756.25	$ 136,872,820.00

SUPPLEMENTARY FIGURE 3: Short term radon testing threshold analysis to determine utility of practice. Panel A: Schematic of SD (sigma) values calculated based on a normal distribution around a reference level of 200 Bq/m^3. Panel B: Using sigma values of 1-5, the number of potential Canadian real estate transactions that would fall outside each confidence interval were calculated based on 2008 to 2018 (data provided by the Canadian Real Estate Foundation). Panel C: Using SD calculated in Figure 2 and Supplementary Figure 1, 3σ values were used to derive thresholds for 5 day alpha track radon test outcomes predictive of a long term (90+ day) alpha track winter test result. Lower thresholds = a reference level minus 1 and then minus 3σ. Upper thresholds = reference level plus 3σ. Cold month (winter) and warm month (summer) values are shown, based on SD derived from data in Figure 2. Panel D: Pie charts showing the predicted outcome of "high", "uncertain" and "low" short term test outcomes based on 578 short term (5 day) alpha track radon tests conducted in winter (March of 2018, average temperature -15°C) in Alberta, and 198 short term (5 day) alpha track radon tests conducted in spring or summer (May to August of 2018, average temperature +15°C) in Alberta. Panel E: Upper graph shows the percentage of annual real estate transactions in either Canada (white bars) or Alberta (grey bars) by month, showing mean daily temperatures (Alberta only). Lower table shows, based on the predicted percent of "high", "uncertain" and "low" short term test outcomes indicated in (H), the projected number of each outcome per year (and associated costs) if every real estate transaction in Canada involved a short term radon test. In Canada, short term radon testing during a real estate transaction costs CAD$125-$400 (industry data provided by the Canadian Association of Radon Scientists and Technologists).
